# To biopsy or not to biopsy a teenager with idiopathic nephrotic syndrome? Biopsy first

**DOI:** 10.1007/s00467-024-06510-6

**Published:** 2024-09-09

**Authors:** Carolina Bigatti, Decimo S. Chiarenza, Andrea Angeletti

**Affiliations:** Nephrology, Dialysis and Transplantation Unit, IRCCS Istituto Giannina Gaslini, Via Gaslini 5, 16147 Genoa, GE Italy

**Keywords:** Idiopathic nephrotic syndrome, Kidney biopsy, Focal segmental glomerulosclerosis, Minimal change disease, Adolescents

## Abstract

Kidney biopsy plays a crucial role in the diagnosis and management of several glomerular diseases. While it is generally considered a routine and safe procedure in children, it should be conducted with the primary objective of addressing the following question: do the prognosis and treatments vary based on the findings of kidney biopsy? In children presenting with idiopathic nephrotic syndrome (INS), guidelines suggest to consider kidney biopsy for individuals older than 12 years, primarily due to the possible increased incidence of different glomerulonephritis compared to younger patients, who predominantly manifest with minimal change disease. However, these guidelines also advocate for uniform therapeutic strategies, typically steroids, irrespective of the age or histological findings. Whether the age of more than 12 years may be a recommendation for performing kidney biopsy at presentation of INS is debatable. Instead, kidney biopsy could be reserved for steroid-resistant cases. On the other hand, when kidney biopsy is performed in INS, particularly in focal segmental glomerulosclerosis, histology may reveal additional lesions, that are strongly associated with a poorer response to treatment and worse clinical outcomes. Therefore, current guidelines on treatments of nephrotic syndrome may appear overly restrictive, despite the relevant findings provided by kidney biopsy. Therefore, in the present manuscript, which is part of a pro–con debate on the management of nephrotic syndrome in adolescents, we emphasize the potential role of performing a kidney biopsy before initiating corticosteroid treatment.

## Introduction

Idiopathic nephrotic syndrome (INS), that manifests with severe proteinuria, hypoalbuminemia, and edema, affects approximately 1–3 individuals per 100,000, contributing to around 10–15% of the kidney failure among children and young adults [1].

Historically, the main histopathological findings in INS include minimal change disease (MCD) and focal and segmental glomerulosclerosis (FSGS), in the absence of glomerular immune deposits. These histological conditions share diffuse foot process effacement and loss of slit diaphragms and may progress to global glomerular sclerosis [2].

In the first decade of life, MCD is the most common histological feature of INS (~ 90% of the patients) [3], followed by FSGS that accounts for most of the remaining cases. The response to steroid treatment is considered to be of greater prognostic value for long-term prognosis than kidney histology [4]; therefore, kidney biopsy is not recommended for INS presenting at this age. On the other hand, current guidelines recommend a kidney biopsy for INS occurring in children older than 12 years old [5–9], mostly due to the increasing frequency of FSGS and other glomerulonephritis, such as membranous nephropathy and membranoproliferative glomerulopathy [8, 10].

Whether the age of more than 12 years may be a recommendation for performing kidney biopsy at presentation of INS is debatable. Previous studies reported that steroid resistance after 6 weeks of treatment at initial presentation is an accurate predictor of FSGS in children with INS, including teenagers, and may be used as the indication for kidney biopsy. This approach would maximize the yield of diagnostic FSGS biopsies while minimizing the number of not needed MCD biopsies [11].

We may reasonably assume that the underlying inquiries regarding the significance of kidney biopsy in teenagers presenting with INS may pertain to the early detection of FSGS as opposed to MCD. More in detail, given the increased rate of FSGS among teenagers presenting with INS, is the early distinction between FSGS and MCD crucial for treatment strategies and prognostic outcomes? Would early FSGS identification enable clinicians to implement more targeted and aggressive therapeutic interventions?

Here, we give an emphasis to the potential role of kidney biopsy in teenagers presenting with INS, with no family history of NS, in enhancing diagnostic precision, tailoring treatment strategies, stratifying risks, prognosticating outcomes, and fostering evidence generation for optimized patient management.

### Kidney pathology of MCD and FSGS

As previously mentioned, MCD and FSGS, both characterized by podocyte injury without immune complex deposits, fall under the category of podocytopathies. In MCD, light microscopy typically reveals either no glomerular lesions or mild focal mesangial prominence. Immunofluorescence may show negative or low-intensity mesangial staining for IgM, sometimes accompanied by C3 or C1q. The hallmark finding of MCD on electron microscopy is extensive foot process effacement, often accompanied by cytoplasmic vacuoles and microvillus transformation, indicative of podocyte injury [12].

FSGS, on the other hand, is a histological pattern that does not largely differentiate between primary, genetic, or secondary forms caused by alterations in glomerular epithelial cells or adaptive changes due to glomerular hypertension. The typical scarring/sclerosis feature is revealed by light microscopy [13].

Immunofluorescence may show non-specific IgM or C3 staining in sclerotic areas, often co-localizing and suggesting complement activation by IgM [14, 15]. Electron microscopy findings, particularly the extent of foot process effacement, may aid in distinguishing primary from maladaptive FSGS, although it is not a reliable marker of genetic forms. While foot process effacement > 80% is suggestive of primary FSGS, cases with a lower percentage of effacement have also been reported [12, 16].

### Other possible histological diagnosis in adolescents presenting with NS

As previously mentioned, kidney biopsy could be considered as part of the initial tests for children older than 12 years, where different causes of NS are more prevalent [9]. The distribution of NS diagnoses varies significantly by age category, and specific patterns can be observed in different age groups, including the 12–18-year-old category. The most common form of NS in infants and young children is MCD, accounting for approximately 90% of cases in children under 10 years old. As children grow older, the distribution of NS subtypes begins to shift. Although less common than MCD and FSGS, membranous nephropathy (MN), characterized by thickening of the glomerular basement membrane due to immune complex deposition, is more frequently diagnosed in adolescents compared to younger children. Also, IgA nephropathy [17, 18], membranoproliferative glomerulonephritis (MPGN) with immune complexes, and C3 glomerulopathy (C3G) can present with NS in adolescents [19], although they are primarily causes of haematuria. Alport kidney disease is characterized by variants in three collagen IV genes, *COL4A3*, *COL4A4*, and *COL4A5*, and typically presents with NS, which includes hematuria, proteinuria, hypertension, and eventual renal insufficiency. However, in approximately 5% of cases, it may present with nephrotic-range proteinuria, occasionally associated with FSGS tip lesions [20, 21]. Lupus nephritis (LN) in adolescents, which can present with NS, typically involves multiple organs and exhibits characteristic laboratory findings [22]. However, renal-limited lupus nephritis refers to a form of glomerulonephritis primarily affecting the kidneys without substantial involvement of other organs or systems. This rare condition is characterized by kidney biopsy findings typical of LN and is observed more commonly in specific regions such as South and East Asia [23].

Therefore, the prognosis and the treatment of NS in adolescents may vary based on the underlying histological diagnosis. While MCD generally has a good prognosis with appropriate treatment, different forms of glomerulonephritis can be more challenging to manage and have a higher risk of progression to chronic kidney disease.

### Is the diagnosis of FSGS useful in deciding treatment strategies?

#### What we already know

Corticosteroids continue to serve as the first-line therapy in INS, inducing disease remission in 80–90% of cases. However, approximately half of patients experience relapse after steroid withdrawal, and about 10–20% develop steroid-resistant INS (SRNS) [5, 9]. In children with steroid sensitivity who receive timely treatment, kidney function is largely preserved, and the disease follows a chronic, relapsing–remitting course that often resolves spontaneously after puberty.

Nonetheless, in 15–25% of cases, INS may manifest during adolescence or adulthood, retaining the characteristic features of childhood-onset INS [24]. These cases typically exhibit a rapid response to corticosteroids upon occurrence, similar to the majority of childhood-onset cases [25].

Therefore, the response to steroid treatment represents the common way to classify INS in children. According to guidelines [5], a majority of children with SRNS and children > 12 years old with steroid sensitivity may receive a kidney biopsy, but the therapeutic approach to pediatric INS remains consistent irrespective of the histopathological pattern. Treatment strategies are primarily guided by the patient’s response to previous corticosteroid therapy rather than specific histological findings. The choice of immunosuppressive medication is often influenced by protocols and clinical considerations, including the frequency of relapses and whether relapses occur while the patient is still on steroid therapy.

Despite the histological lesions, the majority of children and adolescents diagnosed with steroid-sensitive NS achieve remission and maintain normal kidney function. Consequently, the specific histopathological pattern eventually observed in kidney biopsy, whether MCD or FSGS, does not dictate the therapeutic intervention.

#### What we would like to know

In the latest version, the KDIGO guidelines on glomerular diseases dedicated different chapters to INS in children (Chapter 3), to MCD (Chapter 5), and to FSGS (Chapter 6) [5]. All these entities present numerous similarities regarding symptoms and treatment. Steroids represent the first-line treatment in all the three chapters. Based on KDIGO guidelines, the histological characterization does not appear very helpful in the management of the disease. Therefore, categorizing patients into different chapters based on biopsy patterns, despite similar clinical manifestations and treatments which are not contingent on histology, may be reasonably questioned.

In relevant cases, FSGS (less frequently MCD) is characterized by the detection of additional histological lesions which are currently regarded as ancillary findings.

These include the presence of low-intensity mesangial IgM staining, occasionally accompanied by C3 or C1q staining [26]. However, the precise significance of these ancillary histological findings in podocytopathies remains to be fully elucidated.

IgM deposition is reported in 10–35% of INS [27, 28], and it may result from passive entrapment within areas of sclerosis. This hypothesis justifies the IgM deposition in various glomerulopathies [29, 30], but fails to explain co-deposition of complement protein C3 [31] and C1q [32] and the absence of other proteins of similar molecular weight, such as IgG. An alternative theory suggests that IgM binds to specific glomerular epitopes that are either expressed or exposed in response to diverse insults. In essence, glomerular IgM deposition and complement activation may signify a shared final pathway of glomerular injury following toxic, hemodynamic, metabolic, and immunologic insults. Previous studies have demonstrated IgM deposition in patients with pure diffuse mesangial hypercellularity, even in the absence of sclerosis [27]. Moreover, IgM is not uniformly present in globally sclerosed glomeruli.

Observational studies focusing on patients with INS and IgM deposition have highlighted its adverse clinical implications [33, 34]. Specifically, the presence of IgM has been associated with steroid unresponsiveness and a diminished response to standard immunosuppressive therapies [35]. Additionally, IgM deposition in INS has been correlated with the development of impaired kidney function and serves as a predictive marker for progression to kidney failure [36].

In a similar way, C1q deposition is described in around 5% of pediatric patients with FSGS, usually presenting with steroid resistance [32].

Overall, clinical observations suggest that glomerular IgM, C1q, and C3 depositions are associated with more severe disease manifestations and outcomes in patients with INS [31, 37]. However, further researches are warranted to fully understand their pathophysiological relevance and potential implications for implementing a more aggressive therapeutic approach [38].

In summary, current guidelines for the management of podocytopathiesmay appear overly restrictive despite the wealth of clinically relevant information provided by kidney biopsy, particularly in adolescents with INS characterized by the significative increased incidence of FSGS and relative ancillary findings.

#### Is the diagnosis of FSGS useful for the prognosis?

In 1957, Arnold Rich characterized the presence of segmental sclerosis involving juxtamedullary glomeruli in postmortem samples obtained from children who had died from INS [39]. However, it was not until the 1970s that FSGS emerged as a distinct clinicopathologic entity [40]. Since the 1970s, several histologic variants of FSGS have been proposed [13, 41], although there remains disagreement regarding their predictive value [42–44].

While 30–40% of adult patients with FSGS will experience significant kidney insufficiency in the first decade after diagnosis [45], the prognosis of FSGS in childhood appears less clear. Abrantes et al. [46] reported that the incidence of kidney failure in childhood with FSGS is 8%, 17%, and 32% when presenting at 5, 10, and 15 years old, respectively. Similarly, Haas et al. [47] correlated the risk of a worst kidney outcome due to FSGS with the increase of age in children and teenagers.

Therefore, increased efforts have been made to elucidate the characteristics of these different histologic variants and establish a hierarchical classification system that could have an impact on clinical practice. The Columbia Classification of primary FSGS was developed, prioritizing five distinct pathologic variants [13]: (1) Collapsing, (2) tip lesion, (3) cellular, (4) hilar, and (5) not otherwise specified (NOS).

Silverstein et al. [48], in a retrospective analysis on a pediatric cohort, correlated The Columbia Classification with kidney outcome. Results showed that the short-term outcome in pediatric primary FSGS resulted generally favorable, but patients with collapsing FSGS presented a significantly increased risk of chronic kidney disease at 4 years of follow-up.

More recently, Zee et al. [49], in 224 patients with INS (104 in pediatric age) from the Nephrotic Syndrome Study Network (NEPTUNE), investigated 48 histologic (37 glomerular, 9 tubulointerstitial, 2 vascular) and 20 ultrastructural descriptors as predictive of clinical outcomes. Authors applied the so called NEPTUNE Digital Pathology Scoring System (NDPSS), a quantitative and semi-quantitative method. Results demonstrated that the historical approach in differentiating FSGS versus MCD ranked among the top predictors of disease progression and complete remission. They also confirmed that interstitial fibrosis and tubular atrophy strongly predicted worse disease progression, worse proteinuria remission, and worse treatment response, as previously reported [50]. However, the study identified novel features relevant for the prediction of clinical outcomes, such as ultrastructural findings. Ultrastructural reports are usually limited to foot process effacement that poorly correlated with clinical outcomes. On the other hand, Zee et al. [49] identified microvillous transformation and endothelial cell abnormalities as top ultrastructural predictors of clinical outcomes, in line with a previous study from the same group [51]. Briefly, microvillous transformation was linked to a greater rate of remission and treatment response, possibly due to transient alterations in podocyte shape. Moreover, moderate to severe acute tubular injury was associated with a higher probability of treatment response but also elevated risks of disease progression. This suggests that severely nephrotic patients exhibiting microvillous transformation or acute tubular injury may initially respond better to immunosuppression but face poorer long-term outcomes.

Hence, histological analysis may offer valuable insights into identifying patients at an increased risk of progressing to kidney failure. However, according to KDIGO guidelines [5], treatment strategies for such patients often mirror those used for individuals at lower risk. At current, the absence of definitive evidence supporting tailored interventions for histological subtypes largely justifies this uniformity in the therapeutic approach. However, advancements in technology [52, 53] suggest that the time may be right for conducting clinical studies to test diverse therapeutic strategies based on histopathological profiles [54].

#### Histology and possible future perspective in INS

The pathogenesis of INS remains largely elusive [55]. Historically, a T cell disorder resulting in the release of circulating factor(s) increasing the glomerular permeability was believed to be responsible of the disease [56]. However, the identification of this circulating permeability factor(s) still represents a challenge for the nephrology community [57]. Recent findings reported that a subset of patients with INS, mostly diagnosed with MCD, exhibit autoantibodies directed against the podocyte-specific protein NEPHRIN [58, 59]. This discovery suggests potential interconnections between podocyte injury, autoimmune mechanisms, and the response to therapies targeting B cells. Elevated serum anti-NEPHRIN autoantibodies during active disease, associated with the presence of podocyte-associated punctate IgG in kidney biopsies [58], have been described in few cases of native MCD and post-transplant FSGS recurrence [60]. NEPHRIN is an essential structural component of the slit diaphragm, and in vitro and in vivo experiments have confirmed the integrity of NEPHRIN as a fundamental element of glomerular permselectivity [61, 62]. Therefore, anti-NEPHRIN antibodies are postulated to injure glomerular structural and functional integrity, leading to NS.

On the other hand, other reports have questioned the sensitivity and specificity of circulating anti-NEHPRIN antibodies measured with available ELISA assays in correlating with disease activity. This underscores the importance of histology to investigate the kidney deposition in tissues as a marker of the disease, at least for a subgroup of INS.

Several reports have suggested that kidney biopsy facilitates the identification of molecular signatures that can be associated with histopathology and evaluated in relation to clinical outcomes. Among others, Mariani et al. [63] presented an unbiased transcriptomic-driven approach on kidney tissue of patients with either MCD or FSGS across independent, North American, European, and African cohorts. The kidney tissue transcriptomic profile-based clustering molecular profiling identified a subgroup of patients with either MCD or FSGS who shared kidney tumor necrosis factor pathway activation and poor outcomes.

These clinical observations may reveal the heterogeneous nature of the underlying pathogenesis in the current disease classification paradigm. Consequently, the inclusion of patient cohorts with divergent disease etiologies may have contributed to the lack of efficacy in several clinical trials, complicating the development of personalized therapeutic strategies for INS. In the future, histological findings may emerge as pivotal in promptly categorizing subtypes of INS [64].

#### Safety of kidney biopsy and possible drawbacks of “Steroid First”

Percutaneous kidney biopsy is generally considered a safe procedure, particularly after the introduction of spring-loaded needles and real-time imaging [65–69]. The most significant risk associated with a kidney biopsy is hemorrhage, which can range from microscopic hematuria (most common) to severe bleeding necessitating a blood transfusion (a very small percentage of cases). Rarely, surgical intervention is required to manage bleeding. Hematoma formation at the biopsy site is a common occurrence, typically resolving within a few weeks. Pain at the biopsy site is also common but usually subsides within a few hours [67].

This manuscript’s focus does not extend to the discussion of the risks associated with kidney biopsy or the various techniques employed for non-targeted biopsy. However, the daily challenge for physicians worldwide is to determine whether the adverse event profiles of the proposed treatments are acceptable to the patient in their current state of health, both for diagnostic and therapeutical procedures. Therefore, it is strongly encouraged to consider kidney biopsy as a fundamental tool for the diagnosis of kidney diseases. However, each procedure must be carefully balanced with the patient’s condition and the physician’s confidence in performing the procedure.

We recognize that short-term corticosteroid therapy as a first-line treatment in adolescents with INS presents limited risks of adverse events. However, the development of iatrogenic Cushingoid features, steroid-induced acne, and potential growth suppression during this critical pubertal period may be unacceptable to many adolescent patients. Additionally, corticosteroids can precipitate mood disturbances, hyperphagia leading to significant weight gain, and hypertension, further impacting the adolescent’s psychosocial and physical well-being. The risks of steroid-induced hyperglycemia, osteoporosis, and suppression of the hypothalamic–pituitary–adrenal axis are also significant concerns. Therefore, the kidney biopsy before steroids could reduce unnecessary corticosteroid exposure and its adverse effects, enabling a more targeted and less harmful treatment approach.

## Conclusions

Do the treatments for teenagers with INS differ between diagnoses that are made by kidney biopsy? Although kidney biopsy is recommended for individuals older than 12 years presenting with INS, mainly due to the higher incidence of FSGS compared to younger patients who mostly present with MCD, current guidelines advocate uniform therapeutic strategies for all cases of INS, regardless of the age of onset or histological findings.

On the other hand, kidney biopsy may show several histologic and ultrastructural parameters, mostly in FSGS, which are strongly associated with a poorer response to treatment and worse clinical outcomes. It can be argued that such findings may become evident similarly when proteinuria remission is not achieved after 6 weeks of steroid treatment. However, the idea that postponing a more aggressive therapeutic approach in cases of more severe INS has negligible effects on clinical outcomes may be debated [70].

Such findings may suggest the necessity for clinical studies comparing different therapeutic strategies based on histopathological profiles, with the aim to investigate if early identification of some histologic characteristics could enable clinicians to implement more targeted and aggressive therapeutic interventions, sparing several weeks of steroids.

## References

[1] Noone DG, Iijima K, Parekh R (2018) Idiopathic nephrotic syndrome in children. Lancet 392:61–74 29910038 10.1016/S0140-6736(18)30536-1

[2] Maas RJ, Deegens JK, Smeets B, Moeller MJ, Wetzels JF (2016) Minimal change disease and idiopathic FSGS: manifestations of the same disease. Nat Rev Nephrol 12:768–776 27748392 10.1038/nrneph.2016.147

[3] Vivarelli M, Massella L, Ruggiero B, Emma F (2017) Minimal change disease. Clin J Am Soc Nephrol 12:332–345 27940460 10.2215/CJN.05000516 PMC5293332

[4] Gipson DS, Massengill SF, Yao L, Nagaraj S, Smoyer WE, Mahan JD, Wigfall D, Miles P, Powell L, Lin JJ, Trachtman H, Greenbaum LA (2009) Management of childhood onset nephrotic syndrome. Pediatrics 124:747–757 19651590 10.1542/peds.2008-1559

[5] Rovin BH, Adler SG, Barratt J, Bridoux F, Burdge KA, Chan TM, Cook HT, Fervenza FC, Gibson KL, Glassock RJ, Jayne DRW, Jha V, Liew A, Liu ZH, Mejía-Vilet JM, Nester CM, Radhakrishnan J, Rave EM, Reich HN, Ronco P, Sanders JF, Sethi S, Suzuki Y, Tang SCW, Tesar V, Vivarelli M, Wetzels JFM, Lytvyn L, Craig JC, Tunnicliffe DJ, Howell M, Tonelli MA, Cheung M, Earley A, Floege J (2021) Executive summary of the KDIGO 2021 Guideline for the Management of Glomerular Diseases. Kidney Int 100:753–779 34556300 10.1016/j.kint.2021.05.015

[6] Trautmann A, Boyer O, Hodson E, Bagga A, Gipson DS, Samuel S, Wetzels J, Alhasan K, Banerjee S, Bhimma R, Bonilla-Felix M, Cano F, Christian M, Hahn D, Kang HG, Nakanishi K, Safouh H, Trachtman H, Xu H, Cook W, Vivarelli M, Haffner D; International Pediatric Nephrology Association (2023) IPNA clinical practice recommendations for the diagnosis and management of children with steroid-sensitive nephrotic syndrome. Pediatr Nephrol 38:877–919 36269406 10.1007/s00467-022-05739-3 PMC9589698

[7] Ehren R, Benz MR, Brinkkötter PT, Dötsch J, Eberl WR, Gellermann J, Hoyer PF, Jordans I, Kamrath C, Kemper MJ, Latta K, Müller D, Oh J, Tönshoff B, Weber S, Weber LT; German Society for Pediatric Nephrology (2021) Pediatric idiopathic steroid-sensitive nephrotic syndrome: diagnosis and therapy -short version of the updated German best practice guideline (S2e) - AWMF register no. 166–001, 6/2020. Pediatr Nephrol 36:2971–2985 34091756 10.1007/s00467-021-05135-3 PMC8445869

[8] Ehren R, Benz MR, Brinkkötter PT, Dötsch J, Eberl WR, Gellermann J, Hoyer PF, Jordans I, Kamrath C, Kemper MJ, Latta K, Müller D, Oh J, Tönshoff B, Weber S, Weber LT; German Society for Pediatric Nephrology (2021) Commentary on “Pediatric idiopathic steroid-sensitive nephrotic syndrome diagnosis and therapy - short version of the updated German best practice guideline (S2e).” Pediatr Nephrol 36:2961–2966 34091755 10.1007/s00467-021-05136-2 PMC8445862

[9] Vivarelli M, Gibson K, Sinha A, Boyer O (2023) Childhood nephrotic syndrome. Lancet 402:809–824 37659779 10.1016/S0140-6736(23)01051-6

[10] Cravedi P, Angeletti A, Remuzzi G (2017) New biologics in the treatment of rare glomerular diseases of childhood. Curr Opin Pharmacol 33:27–33 28456094 10.1016/j.coph.2017.03.010

[11] Alshami A, Roshan A, Catapang M, Jöbsis JJ, Kwok T, Polderman N, Sibley J, Sibley M, Mammen C, Matsell DG; Pediatric Nephrology Clinical Pathway Development Team (2017) Indications for kidney biopsy in idiopathic childhood nephrotic syndrome. Pediatr Nephrol 32:1897–1905 28540445 10.1007/s00467-017-3687-3

[12] Fogo AB, Lusco MA, Najafian B, Alpers CE (2015) AJKD atlas of renal pathology: minimal change disease. Am J Kidney Dis 66:376–377 26210726 10.1053/j.ajkd.2015.04.006

[13] D’Agati VD, Fogo AB, Bruijn JA, Jennette JC (2004) Pathologic classification of focal segmental glomerulosclerosis: a working proposal. Am J Kidney Dis 43:368–382 14750104 10.1053/j.ajkd.2003.10.024

[14] Strassheim D, Renner B, Panzer S, Fuquay R, Kulik L, Ljubanović D, Holers VM, Thurman JM (2013) IgM contributes to glomerular injury in FSGS. J Am Soc Nephrol 24:393–406 23393315 10.1681/ASN.2012020187 PMC3582199

[15] Panzer SE, Laskowski J, Renner B, Kulik L, Ljubanovic D, Huber KM, Zhong W, Pickering MC, Holers VM, Thurman JM (2015) IgM exacerbates glomerular disease progression in complement-induced glomerulopathy. Kidney Int 88:528–537 25945405 10.1038/ki.2015.120 PMC4556608

[16] Zamami R, Kohagura K, Kinjyo K, Nakamura T, Kinjo T, Yamazato M, Ishida A, Ohya Y (2021) The association between glomerular diameter and secondary focal segmental glomerulosclerosis in chronic kidney disease. Kidney Blood Press Res 46:433–440 34315152 10.1159/000515528

[17] Jiang Y, Chen P, Zhao W, Liu L, Shi S, Lv J, Zhang H (2024) Distinct characteristics and prognosis of IgA nephropathy patients with nephrotic syndrome: a propensity score-matched cohort study. Front Med (Lausanne) 11:1344219 38439903 10.3389/fmed.2024.1344219 PMC10910015

[18] Hogg RJ (2010) Idiopathic immunoglobulin A nephropathy in children and adolescents. Pediatr Nephrol 25:823–829 19194728 10.1007/s00467-008-1096-3 PMC2839527

[19] Spartà G, Gaspert A, Neuhaus TJ, Weitz M, Mohebbi N, Odermatt U, Zipfel PF, Bergmann C, Laube GF (2018) Membranoproliferative glomerulonephritis and C3 glomerulopathy in children: change in treatment modality? A report of a case series. Clin Kidney J 11:479–490 30094012 10.1093/ckj/sfy006 PMC6070093

[20] Frascà GM, Onetti-Muda A, Mari F, Longo I, Scala E, Pescucci C, Roccatello D, Alpa M, Coppo R, Li Volti G, Feriozzi S, Bergesio F, Schena FP, Renieri A; Italian Renal Immunopathology Group (2005) Thin glomerular basement membrane disease: clinical significance of a morphological diagnosis–a collaborative study of the Italian Renal Immunopathology Group. Nephrol Dial Transplant 20:545–551 15618242 10.1093/ndt/gfh617

[21] Savige J, Rana K, Tonna S, Buzza M, Dagher H, Wang YY (2003) Thin basement membrane nephropathy. Kidney Int 64:1169–1178 12969134 10.1046/j.1523-1755.2003.00234.x

[22] Ruggiero B, Vivarelli M, Gianviti A, Benetti E, Peruzzi L, Barbano G, Corona F, Ventura G, Pecoraro C, Murer L, Ghiggeri GM, Pennesi M, Edefonti A, Coppo R, Emma F (2013) Lupus nephritis in children and adolescents: results of the Italian Collaborative Study. Nephrol Dial Transplant 28:1487–1496 23345627 10.1093/ndt/gfs589

[23] Huerta A, Bomback AS, Liakopoulos V, Palanisamy A, Stokes MB, D’Agati VD, Radhakrishnan J, Markowitz GS, Appel GB (2012) Renal-limited ‘lupus-like’ nephritis. Nephrol Dial Transplant 27:2337–2342 22207326 10.1093/ndt/gfr663

[24] Tullus K (2023) Why FSGS keeps being presented as a disease although it is not. Nephrol Dial Transplant 38:2426–2427 37355785 10.1093/ndt/gfad128

[25] Mirioglu S, Daniel-Fischer L, Berke I, Ahmad SH, Bajema IM, Bruchfeld A, Fernandez-Juarez GM, Floege J, Frangou E, Goumenos D, Griffith M, Moran SM, van Kooten C, Steiger S, Stevens KI, Turkmen K, Willcocks LC, Kronbichler A (2024) Management of adult patients with podocytopathies: an update from the ERA Immunonephrology Working Group. Nephrol Dial Transplant 39:569–580 38341276 10.1093/ndt/gfae025 PMC11024823

[26] Angeletti A, Reyes-Bahamonde J, Cravedi P, Campbell KN (2017) Complement in Non-Antibody-Mediated Kidney Diseases. Front Med (Lausanne) 4:99 28748184 10.3389/fmed.2017.00099 PMC5506082

[27] Habib R, Girardin E, Gagnadoux MF, Hinglais N, Levy M, Broyer M (1988) Immunopathological findings in idiopathic nephrosis: clinical significance of glomerular “immune deposits.” Pediatr Nephrol 2:402–408 3153051 10.1007/BF00853431

[28] Gephardt GN, Tubbs RR, Popowniak KL, McMahon JT (1986) Focal and segmental glomerulosclerosis. Immunohistologic study of 20 renal biopsy specimens. Arch Pathol Lab Med 110:902–905 2429634

[29] Mujais SK, Emmanouel DS, Kasinath BS, Spargo BH (1985) Marked proteinuria in hypertensive nephrosclerosis. Am J Nephrol 5:190–195 3160240 10.1159/000166931

[30] Ainsworth SK, Hirsch HZ, Brackett NC Jr, Brissie RM, Williams AV Jr, Hennigar GR (1982) Diabetic glomerulonephropathy: histopathologic, immunofluorescent, and ultrastructural studies of 16 cases. Hum Pathol 13:470–478 7042531 10.1016/s0046-8177(82)80030-0

[31] Peng Y, Li B, Li X, Ju T, Zhang Z, Wang P, Sun T, Shu J, Wang M, Sun X, Chen H, Gao C, Xia Z (2023) Glomerular capillary C3 deposition as a risk factor for unfavorable renal outcome in pediatric primary focal segmental glomerular sclerosis. Front Pediatr 11:1137375 37025292 10.3389/fped.2023.1137375 PMC10070806

[32] Peng Y, Ju T, Gao C, Xia Z, Wang M, Sun X, Wang R, Li X, Wei Y, Jia L, Chen H (2023) A clinicopathological and prognostic study of 18 children with C1q nephropathy and focal segmental glomerulosclerosis: an 18-year experience from a single center. J Nephrol 36:1615–1625 37428438 10.1007/s40620-023-01679-9

[33] Xiong L, Liu L, Tao Y, Guo H (2023) Clinical significance of IgM and C3 deposition in children with primary immunoglobulin A nephropathy. J Nephrol 36:2213–2222 37542609 10.1007/s40620-023-01724-7

[34] Silverstein DM, Craver RD (2008) Mesangial hypercellularity in children: presenting features and outcomes. Pediatr Nephrol 23:921–928 18324424 10.1007/s00467-008-0750-0

[35] Swartz SJ, Eldin KW, Hicks MJ, Feig DI (2009) Minimal change disease with IgM+ immunofluorescence: a subtype of nephrotic syndrome. Pediatr Nephrol 24:1187–1192 19219463 10.1007/s00467-009-1130-0

[36] Myllymäki J, Saha H, Mustonen J, Helin H, Pasternack A (2003) IgM nephropathy: clinical picture and long-term prognosis. Am J Kidney Dis 41:343–350 12552495 10.1053/ajkd.2003.50042

[37] Angeletti A, Cantarelli C, Petrosyan A, Andrighetto S, Budge K, D’Agati VD, Hartzell S, Malvi D, Donadei C, Thurman JM, Galešić-Ljubanović D, He JC, Xiao W, Campbell KN, Wong J, Fischman C, Manrique J, Zaza G, Fiaccadori E, La Manna G, Fribourg M, Leventhal J, Da Sacco S, Perin L, Heeger PS, Cravedi P (2020) Loss of decay-accelerating factor triggers podocyte injury and glomerulosclerosis. J Exp Med 217:e20191699 32717081 10.1084/jem.20191699 PMC7478737

[38] Angeletti A, Magnasco A, AntonellaTrivelli, Degl’Innocenti LM, Piaggio G, Lugani F, Caridi G, Verrina E, Cravedi P, Ghiggeri GM (2022) Refractory minimal change disease and focal segmental glomerular sclerosis treated with anakinra. Kidney Int Rep 7:121–124 35005321 10.1016/j.ekir.2021.10.018 PMC8720665

[39] Rich AR (1957) A hitherto undescribed vulnerability of the juxtamedullary glomeruli in lipoid nephrosis. Bull Johns Hopkins Hosp 100:173–186 13426687

[40] Churg J, Habib R, White RH (1970) Pathology of the nephrotic syndrome in children: a report for the International Study of Kidney Disease in Children. Lancet 760:1299–1302 4193942 10.1016/s0140-6736(70)91905-7

[41] Thomas DB, Franceschini N, Hogan SL, Ten Holder S, Jennette CE, Falk RJ, Jennette JC (2006) Clinical and pathologic characteristics of focal segmental glomerulosclerosis pathologic variants. Kidney Int 69:920–926 16518352 10.1038/sj.ki.5000160

[42] Schwartz MM, Korbet SM, Rydell J, Borok R, Genchi R (1995) Primary focal segmental glomerular sclerosis in adults: prognostic value of histologic variants. Am J Kidney Dis 25:845–852 7771480 10.1016/0272-6386(95)90566-9

[43] (1985) Focal segmental glomerulosclerosis in children with idiopathic nephrotic syndrome. A report of the Southwest pediatric nephrology study group. Kidney Int 27:442–9. 10.1038/ki.1985.29 10.1038/ki.1985.29 3886999

[44] Howie AJ, Pankhurst T, Sarioglu S, Turhan N, Adu D (2005) Evolution of nephrotic-associated focal segmental glomerulosclerosis and relation to the glomerular tip lesion. Kidney Int 67:987–1001 15698437 10.1111/j.1523-1755.2005.00162.x

[45] Ichikawa I, Fogo A (1996) Focal segmental glomerulosclerosis. Pediatr Nephrol 10:374–391 8792409 10.1007/BF00866790

[46] Abrantes MM, Cardoso LS, Lima EM, Silva JM, Diniz JS, Bambirra EA, Oliveira EA (2006) Clinical course of 110 children and adolescents with primary focal segmental glomerulosclerosis. Pediatr Nephrol 21:482–489 16520952 10.1007/s00467-006-0019-4

[47] Dragovic D, Rosenstock JL, Wahl SJ, Panagopoulos G, DeVita MV, Michelis MF (2005) Increasing incidence of focal segmental glomerulosclerosis and an examination of demographic patterns. Clin Nephrol 63:1–7 15678691 10.5414/cnp63001

[48] Silverstein DM, Craver R (2007) Presenting features and short-term outcome according to pathologic variant in childhood primary focal segmental glomerulosclerosis. Clin J Am Soc Nephrol 2:700–707 17699485 10.2215/CJN.00230107

[49] Zee J, Liu Q, Smith AR, Hodgin JB, Rosenberg A, Gillespie BW, Holzman LB, Barisoni L, Mariani LH; Nephrotic Syndrome Study Network (NEPTUNE); NEPTUNE Members (2022) Kidney biopsy features most predictive of clinical outcomes in the spectrum of minimal change disease and focal segmental glomerulosclerosis. J Am Soc Nephrol 33:1411–1426 35581011 10.1681/ASN.2021101396 PMC9257823

[50] Mariani LH, Martini S, Barisoni L, Canetta PA, Troost JP, Hodgin JB, Palmer M, Rosenberg AZ, Lemley KV, Chien HP, Zee J, Smith A, Appel GB, Trachtman H, Hewitt SM, Kretzler M, Bagnasco SM (2018) Interstitial fibrosis scored on whole-slide digital imaging of kidney biopsies is a predictor of outcome in proteinuric glomerulopathies. Nephrol Dial Transplant 33:310–318 28339906 10.1093/ndt/gfw443 PMC5837529

[51] Royal V, Zee J, Liu Q, Avila-Casado C, Smith AR, Liu G, Mariani LH, Hewitt S, Holzman LB, Gillespie BW, Hodgin JB, Barisoni L (2020) Ultrastructural characterization of proteinuric patients predicts clinical outcomes. J Am Soc Nephrol 31:841–854 32086276 10.1681/ASN.2019080825 PMC7191920

[52] Schena FP, Magistroni R, Narducci F, Abbrescia DI, Anelli VW, Di Noia T (2022) Artificial intelligence in glomerular diseases. Pediatr Nephrol 37:2533–2545 35266037 10.1007/s00467-021-05419-8

[53] Nadkarni GN, Chaudhary K, Coca SG (2019) Machine learning in glomerular diseases: promise for precision medicine. Am J Kidney Dis 74:290–292 31200978 10.1053/j.ajkd.2019.04.011

[54] Duret LC, Hamidouche T, Steers NJ, Pons C, Soubeiran N, Buret D, Gilson E, Gharavi AG, D’Agati VD, Shkreli M (2024) Targeting WIP1 phosphatase promotes partial remission in experimental collapsing glomerulopathy. Kidney Int 105:980–996 38423182 10.1016/j.kint.2024.02.009

[55] Floege J, Amann K (2016) Primary glomerulonephritides. Lancet 387:2036–2048 26921911 10.1016/S0140-6736(16)00272-5

[56] Gallon L, Leventhal J, Skaro A, Kanwar Y, Alvarado A (2012) Resolution of recurrent focal segmental glomerulosclerosis after retransplantation. N Engl J Med 366:1648–1649 22533598 10.1056/NEJMc1202500

[57] Angeletti A, Bruschi M, Kajana X, La Porta E, Spinelli S, Caridi G, Lugani F, Verrina EE, Ghiggeri GM (2023) Biologics in steroid resistant nephrotic syndrome in childhood: review and new hypothesis-driven treatment. Front Immunol 14:1213203 37705972 10.3389/fimmu.2023.1213203 PMC10497215

[58] Watts AJB, Keller KH, Lerner G, Rosales I, Collins AB, Sekulic M, Waikar SS, Chandraker A, Riella LV, Alexander MP, Troost JP, Chen J, Fermin D, Yee JL, Sampson MG, Beck LH Jr, Henderson JM, Greka A, Rennke HG, Weins A (2022) Discovery of autoantibodies targeting nephrin in minimal change disease supports a novel autoimmune etiology. J Am Soc Nephrol 33:238–252 34732507 10.1681/ASN.2021060794 PMC8763186

[59] Chebotareva N, Vinogradov A, Birukova Y, Alentov I, Sergeeva N, Chemodanova D, Kononikhin AS, Moiseev SV (2024) A pilot study of anti-nephrin antibodies in podocytopaties among adults. Nephrology (Carlton) 29:86–92 37864506 10.1111/nep.14249

[60] Shirai Y, Miura K, Ishizuka K, Ando T, Kanda S, Hashimoto J, Hamasaki Y, Hotta K, Ito N, Honda K, Tanabe K, Takano T, Hattori M (2024) A multi-institutional study found a possible role of anti-nephrin antibodies in post-transplant focal segmental glomerulosclerosis recurrence. Kidney Int 105:608–617 38110152 10.1016/j.kint.2023.11.022

[61] Grahammer F, Schell C, Huber TB (2013) Molecular understanding of the slit diaphragm. Pediatr Nephrol 28:1957–1962 23233041 10.1007/s00467-012-2375-6

[62] Grahammer F, Schell C, Huber TB (2013) The podocyte slit diaphragm–from a thin grey line to a complex signalling hub. Nat Rev Nephrol 9:587–598 23999399 10.1038/nrneph.2013.169

[63] Mariani LH, Eddy S, AlAkwaa FM et al (2023) Precision nephrology identified tumor necrosis factor activation variability in minimal change disease and focal segmental glomerulosclerosis. Kidney Int 103:565–579 36442540 10.1016/j.kint.2022.10.023 PMC10347421

[64] Muhlig AK, Oh J, Huber TB (2023) Precision nephrology: from molecular diagnostics to an individualized therapy. Kidney Int 103:464–466 36822751 10.1016/j.kint.2022.12.017

[65] Doyle AJ, Gregory MC, Terreros DA (1994) Percutaneous native renal biopsy: comparison of a 1.2-mm spring-driven system with a traditional 2-mm hand-driven system. Am J Kidney Dis 23:498–503 8154484 10.1016/s0272-6386(12)80370-2

[66] Nyman RS, Cappelen-Smith J, al Suhaibani H, Alfurayh O, Shakweer W, Akhtar M, (1997) Yield and complications in percutaneous renal biopsy. A comparison between ultrasound-guided gun-biopsy and manual techniques in native and transplant kidneys. Acta Radiol 38:431–436 9191436 10.1080/02841859709172096

[67] MacGinley R, Champion De Crespigny PJ, Gutman T, Lopez-Vargas P, Manera K, Menahem S, Saunders J, See E, Voss D, Wong J (2019) KHA-CARI Guideline recommendations for renal biopsy. Nephrology (Carlton) 24:1205–1213 31490584 10.1111/nep.13662

[68] Hogan JJ, Mocanu M, Berns JS (2016) The native kidney biopsy: update and evidence for best practice. Clin J Am Soc Nephrol 11:354–362 26339068 10.2215/CJN.05750515 PMC4741037

[69] Korbet SM, Volpini KC, Whittier WL (2014) Percutaneous renal biopsy of native kidneys: a single-center experience of 1,055 biopsies. Am J Nephrol 39:153–162 24526094 10.1159/000358334

[70] Hunley TE, Yared A, Fogo A, MacDonell RC Jr (1998) Nephrotic syndrome in an adolescent: the cry of the wolf. Am J Kidney Dis 31:155–160 9428468 10.1053/ajkd.1998.v31.pm9428468

